# Single-incision laparoscopic total colectomy using an organ retractor: A case report

**DOI:** 10.1016/j.amsu.2020.04.032

**Published:** 2020-05-20

**Authors:** Kazuhide Ishimaru, Tetsuro Tominaga, Takashi Nonaka, Makoto Hisanaga, Akiko Fukuda, Takafumi Yoshimoto, Daiki Takei, Shigekazu Hidaka, Terumitsu Sawai, Takeshi Nagayasu

**Affiliations:** a Departments of Surgical Oncology, Nagasaki University Graduate School of Biomedical Sciences, 1-7-1 Sakamoto, Nagasaki, 852-8501, Japan; b Cardiopulmonary Rehabilitation Science, Nagasaki University Graduate School of Biomedical Sciences, 1-7-1 Sakamoto, Nagasaki, 852-8501, Japan

**Keywords:** Total colectomy, Organ retractor, Single-incision laparoscopic surgery, SILS, single-port laparoscopic surgery

## Abstract

**Introduction:**

Single-incision laparoscopic surgery (SILS) has benefits, including less postoperative pain, a shorter incision, and improved cosmesis. However, SILS is technically difficult because of the limited movement. An organ retractor is an instrument that has the potential to overcome these limitations.

**Presentation of case:**

An 85-year-old woman with hematochezia was referred to our hospital. Emergency endoscopy showed diverticulosis of the entire colon and active bleeding from the ascending colon. Despite endoscopic clipping, the bleeding continued. SILS total colectomy using an organ retractor was performed due to uncontrollable diverticular bleeding. A 3-cm incision was placed in the umbilicus, and three conventional ports were inserted into the single umbilical incision. An organ retractor was used for hepatocolic ligament transection, transection of the ileocolic vessels, and transection of the mesentery of the sigmoid colon. For each transection, the tension was adjusted to provide a good operative view. The patient's postoperative course was uneventful.

**Conclusion:**

An organ retractor was effective for SILS total colectomy to maintain an adequate operative view, which enabled safe dissection.

## Highlights

• Total colectomy causes severe damage because of the extensive organ mobilization.
• SILS has a benefit in terms of faster postoperative recovery.
• SILS is often challenging because of the limited movement.
• An organ retractor is effective for overcoming these restrictions.

## 1 Introduction

Total colectomy is often performed for patients with ulcerative colitis and familial adenomatous polyposis [1,2]. However, this procedure causes severe damage because of the extensive organ mobilization, long operation time, and large blood loss [3,4]. Laparoscopic surgery has been the standard procedure for colorectal disease, and it has benefit in terms of faster postoperative recovery and shorter hospital stay [5]. Recently, there have been several reports of the safety and feasibility of laparoscopic total colectomy with the advantage of minimal invasiveness [6,7]. Single-incision laparoscopic surgery (SILS) is the latest innovation in minimally invasive surgery, and it reduces the risk of trocar-related complications, requires shorter incisions, reduces postoperative pain, and improves cosmesis compared to conventional laparoscopic surgery [8,9]. However, SILS is often challenging and has some limitations, because of the restricted movement of the surgical device, with loss of triangulation that could lead to an inadequate operative field [10,11]. In cases of total colectomy, maintaining an adequate operative field is more difficult because a multi-dimensional approach is needed for bowel mobilization.

An internal organ retractor (B. Braun, Tokyo, Japan) is a peg-like device developed for softly and gently grasping organs or tissue. We previously reported the effectiveness of SILS-right colectomy using an organ retractor for overcoming these restrictions [12]. In various operations, including lobectomy [13], esophagectomy [14], and adrenalectomy [15], an organ retractor enabled not only maintenance of an adequate operative field, but also the prevention of surrounding tissue injury.

In the present case report, the case of a patient with diverticulosis of the entire colon treated with SILS total colectomy using an organ retractor is presented. This work has been reported in line with the SCARE criteria [16].

## 2 Presentation of case

**Fig. 1:**
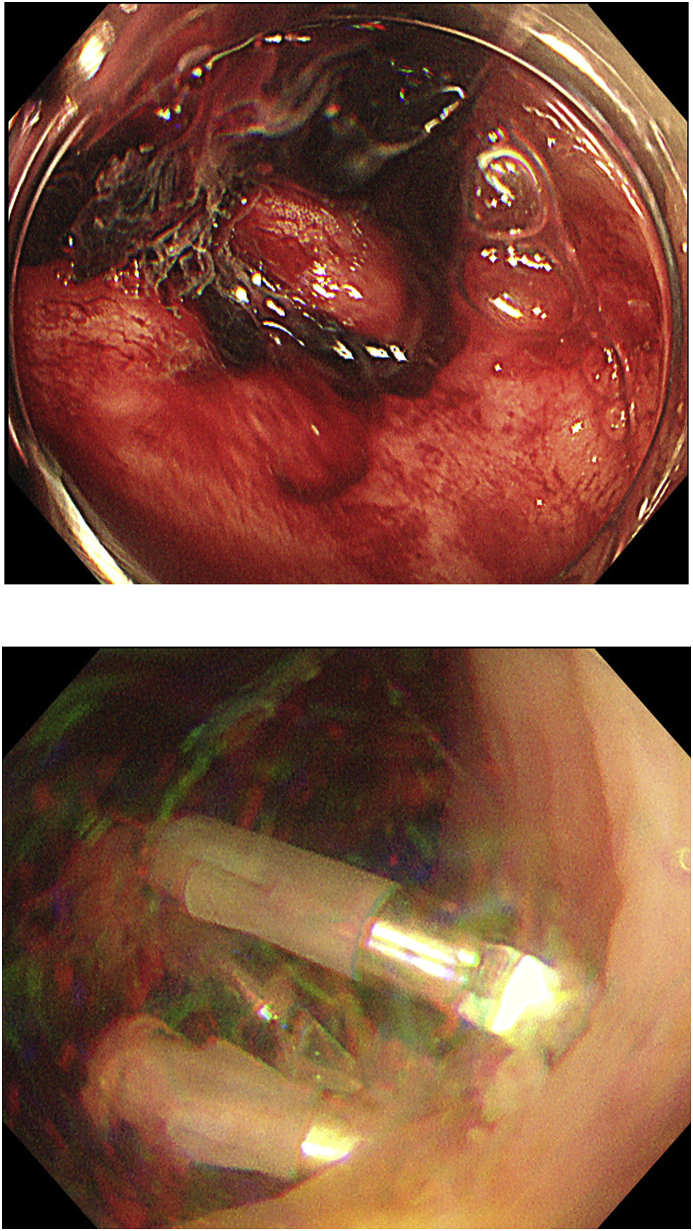
Emergency endoscopy showed diverticulosis of the entire colon. Diverticular bleeding from the ascending colon was suspected (Fig. 1a), and endoscopic clipping was performed (Fig. 1b).

**Fig. 2:**
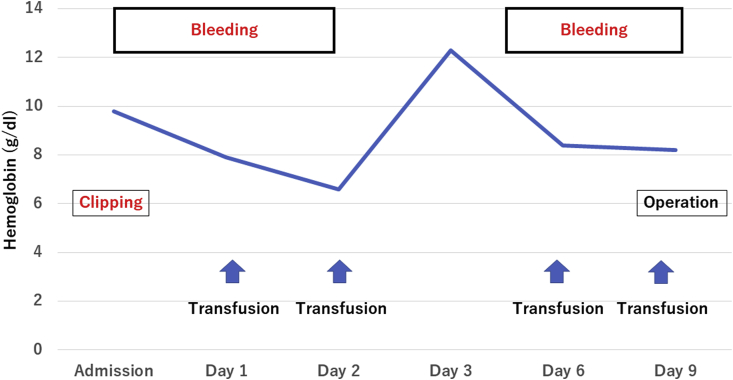
Clinical course of the patient before operation.

An 85-year-old woman with hematochezia was brought to our hospital emergency department by ambulance. She had no abdominal pain or other digestive symptoms. Her physical examination showed severe anemia with facial pallor, but there was no tenderness or distention of the abdomen. She had previously undergone coronary stenting due to myocardial infarction and was on anticoagulant drug therapy. She had also had a hysterectomy for a uterine myoma. Laboratory data showed severe anemia. CT angiography could not detect extravasation from colonic arteries. Emergency endoscopy showed diverticulosis of the entire colon (Fig. 1a), with active diverticular bleeding from the ascending colon, and endoscopic clipping was performed (Fig. 1b). Esophago-gastro-duodenoscopy could not demonstrate massive bleeding from the gastrointestinal tract. After treatment, the hematochezia decreased at two days, but from 5 days after admission, the patient had continuous uncontrollable bleeding. Colonoscopy after re-bleeding could not identify the exact bleeding site. She became hemodynamically unstable. The patient was finally diagnosed with multiple diverticular bleeding of the entire colon, and total colectomy was performed using SILS technique (Fig. 2).

**Fig. 3:**
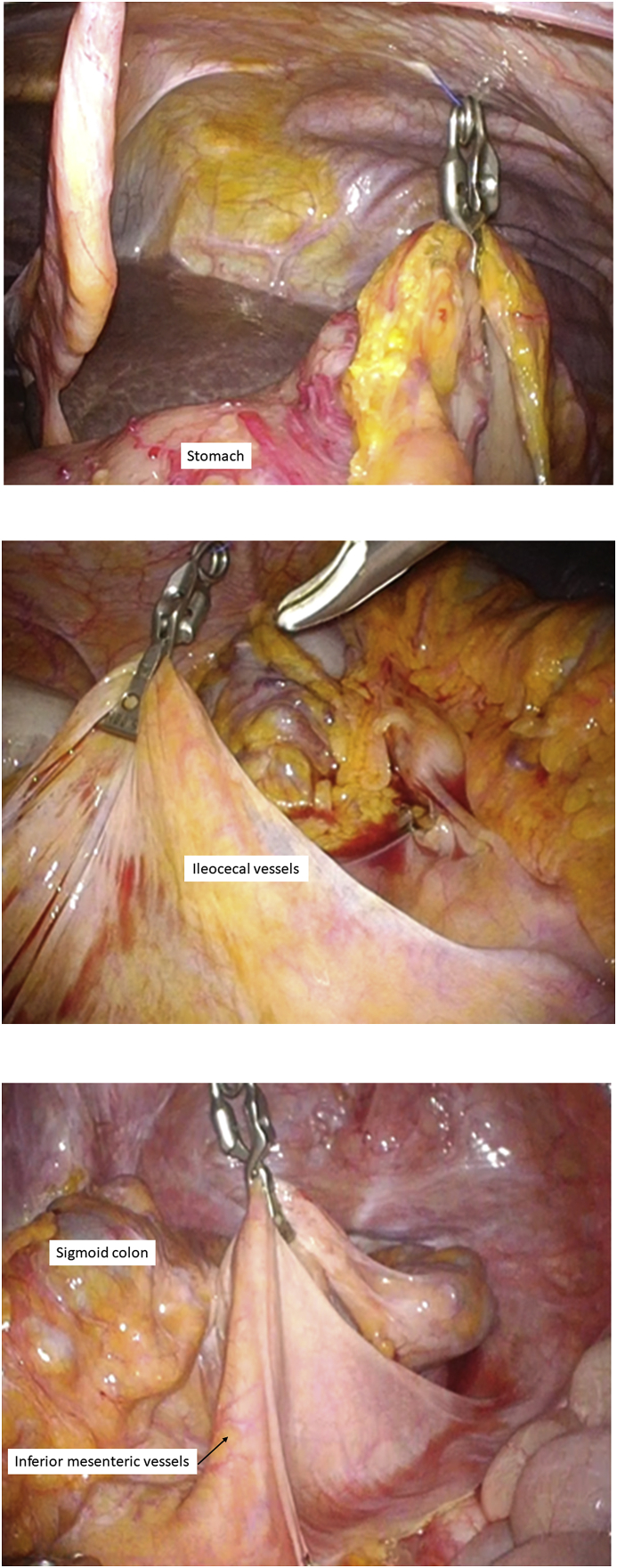
The posterior wall of the stomach was grasped using an organ retractor to maintain a good view of the hepatic flexure (Fig. 3a), and the pedicle of the ileocecal artery and vein was grasped (Fig. 3b).

**Fig. 4:**
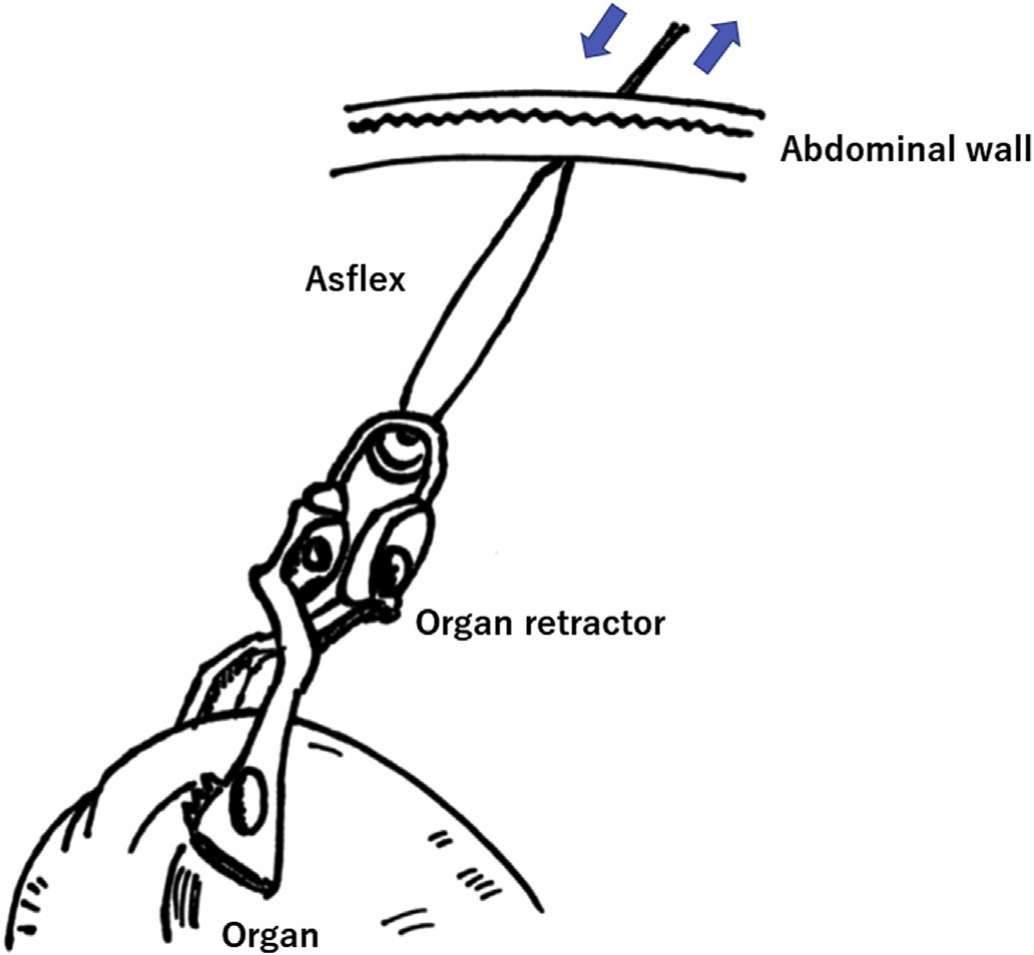
An organ retractor was controlled to grasp the organ in an appropriate position. Then the tail of the organ retractor was pulled using Asflex, which was inserted extracorporeally. Organ tension could be controlled by adjusting the tension of the Asflex.

A zigzag incision was placed in the umbilicus. Then, EZ access (Hakko-medical, Tokyo, Japan) was inserted through the wound, and one 12-mm port and two 5-mm ports were placed, one for the scope and two for the energy device. First, mobilization was started from the right-side colon by the medial approach. Following opening of the omental bursa, the posterior wall of the stomach was grasped using an organ retractor to maintain a good view of the hepatic flexure, and the hepatic flexure was mobilized (Fig. 3a). Then, the pedicle of the ileocecal artery and vein was grasped by the organ retractor (Fig. 3b). After the vessels were transected, mobilization of the right-side colon was completed by dissection of the lateral attachment. The tail of the organ retractor was pulled using Asflex (Crownjun, Chiba, Japan) (Fig. 4).

**Fig. 5:**
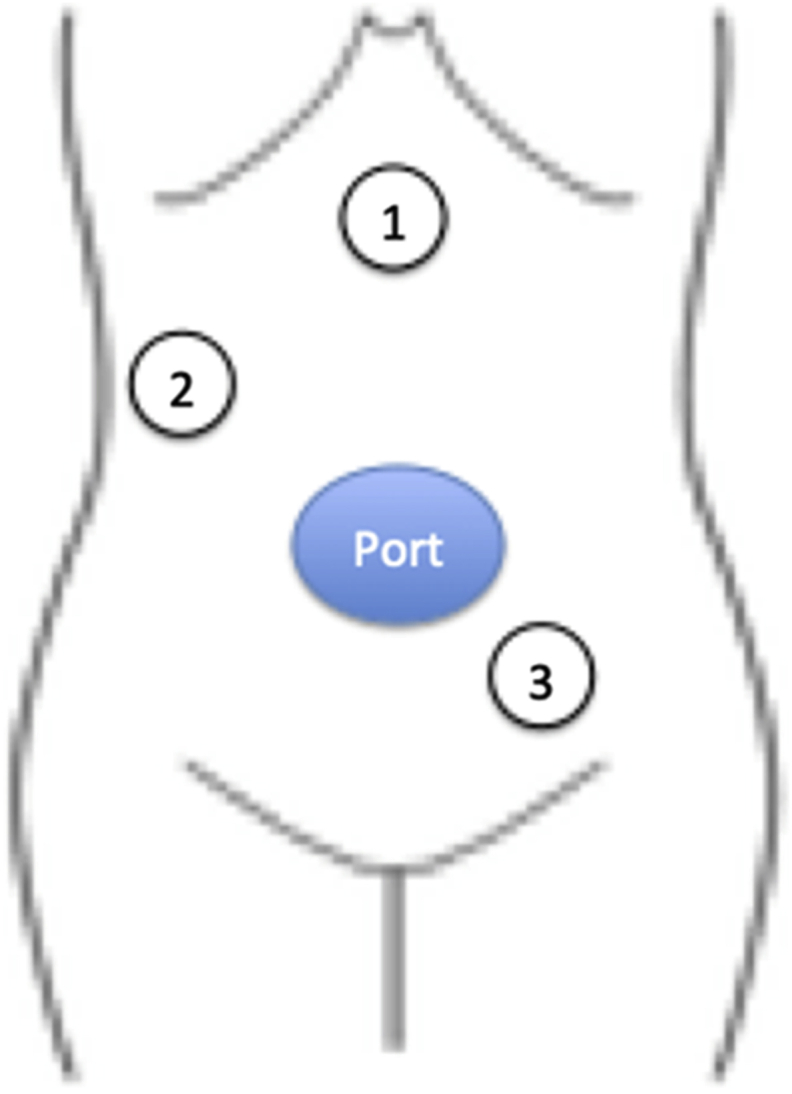
The Asflex insertion points. When the hepatic or splenic flexure was mobilized, the Asflex was inserted from part 1. When the ileocecal vessels was transected, the Asflex was inserted from part 2. When the inferior mesenteric vessels were transected, the Asflex was inserted from part 3.

Next, the splenic ligament and inferior mesenteric vessels were transected. The mesentery of the sigmoid colon was then grasped using an organ retractor (Fig. 3c), and the transection of the inferior mesenteric artery and full mobilization of the colon and rectum above the peritoneal reflection were completed. For each transection, the insertion points of the Asflex were changed to provide a good operative view (Fig. 5). The operator's position was on the left side of the patient when the right-side colon was mobilized, and then switched to the opposite side when the left-side colon was handled. To remove the specimen from the body, the wound was dilated to 5 cm. The lesion was then transected by Endo GIA purple 60 from the 12-mm port (Covidien, Minneapolis, MN, USA). An ileal pouch was made, and ileorectal anastomosis was performed with double stapling technique. The operation time was 194 min, and intraoperative blood loss was 30 ml. The patient's postoperative course was good.

## 3 Discussion

In the present case report, the effectiveness of an organ retractor for SILS total colectomy for maintaining an adequate operative field was described.

Gastrointestinal bleeding is a medical emergency, and its mortality has reached 40% in elderly patients with some comorbidities [17]. Colonoscopy has been reported to locate the bleeding site in 70% of cases, but it has some disadvantages because it needs preparation [18]. CT angiography is another diagnostic technique for evaluating intestinal bleeding as a minimally invasive tool [19]. A meta-analysis evaluating the accuracy of CT angiography in the diagnosis of intestinal bleeding showed that overall sensitivity was 85.2%, and overall specificity was 92.1%. However, the diagnosis was sometimes affected by severe active bleeding. In the present case, the exact location of bleeding could not be identified by colonoscopy and CT angiography.

Several controlled studies and randomized, controlled trials have shown that SILS is a safe and feasible procedure compared to conventional laparoscopic surgery [9,20,21]. However, SILS is still technically challenging owing to instrument crowding, loss of triangulation and in-line viewing, and it requires a high level of skill [10,11]. To overcome these restrictions, the application of single-port plus one-port laparoscopic surgery (SILS+1) has recently gained increasing attention from colorectal surgeons [10,11,[22], [23], [24], [25], [26]]. Kawahara et al. reported the safety of SILS+1 for partial colectomy. In performing SILS for transverse colon cancer, safe transection of both middle colic vessels and the gastrocolic trunk is crucial. It is sometimes very difficult even for skillful surgeons when there are anomalies. They argued that the energy device could be inserted in the best position using an additional port [22]. There have been several reports of the SILS+1 approach for sigmoidectomy or anterior transection [[25], [26], [27]]. The main difficulty of the SILS approach to the procedures is the transection of the rectum with a stapler, because, when the stapler is inserted through the umbilical port, its position is parallel to the rectum [11,23]. They placed an additional port in the right lower quadrant when performing these procedures and resected the rectum safely. Total colectomy requires a multi-dimensional approach, but the reports of SILS-total colectomy are still limited [28,29]. In regard to total colectomy, only one additional port could be insufficient to obtain an adequate operative view, because we should mobilize the entire colon including the right-side colon, liver flexure, splenic flexure, and left-side colon.

In the present case, an organ retractor was used to overcome loss of triangulation, which could have led to an inadequate operative field. The instrument could be removed and adapted to change the retraction as the surgery progressed in various situations, and an adequate operative view, the same as obtained using the SILS+1 approach, could be maintained. Furthermore, organ tension could be changed by adjusting the organ retractor with Asflex, which controlled the continuous traction needed for the dissection line and enabled the operation to proceed smoothly (Fig. 4). Indeed, it was used for transection of the hepatocolic ligament, the ileocecal vessels, the insertion of the mesentery proper, and the inferior mesenteric vessels; no additional port was required to complete both transection of the root vessels and total colonic mobilization.

SILS has been reported to be associated with better perioperative outcomes, including shorter operation time, less blood loss, and shorter hospital stay, compared to conventional laparoscopic surgery [30]. Konishi et al. examined the feasibility and morbidity of laparoscopic total colectomy using Japanese multicenter data [2]. They showed that the operation time and blood loss of laparoscopic total colectomy with ileorectal anastomosis were 313 min (175–682 min) and 65 ml (0–920 ml), respectively. In the present case, operation time was 195 min, and blood loss was 30 ml, which were reasonable compared to the previous report.

The SILS approach has a longer learning curve compared to the SILS+1 approach or conventional laparoscopic surgery [23]. Li et al. argued that SILS+1 could overcome device interference or help maintain the field [24]. The operative procedure of SILS+1 is similar to that of conventional laparoscopic surgery; therefore, the transition from conventional laparoscopic surgery to SILS+1 was relatively easy. However, insertion of an additional port could risk herniation or injury of the intestine. We think that an organ retractor has the potential to provide the same advantage as SILS+1 technique without the additional wound. Furthermore, the organ retractor could grasp and release in several positions to provide an adequate view, which could be very instructive for younger surgeons when considering how to create a good operative view.

## 4 Conclusion

A case of single-incision laparoscopic total colectomy using an organ retractor was reported. An organ retractor is effective and helpful for SILS to maintain a good operative view to mobilize the entire colon.

## Ethical approval

Not applicable.

## Funding

This research did not receive any specific grant from funding agencies in the public, commercial, or not-for-profit sectors.

## Author contributions

Kazuhide Ishimaru, Tetsuro Tominaga, and Takashi Nonaka developed the study concept. Makoto Hisanaga, Akiko Fukuda, Takafumi Yoshimoto, Daiki Takei, and Shigekazu Hidaka collaborated in the patient's care. Terumitsu Sawai and Takeshi Nagayasu provided input on the manuscript.

## Trial registry number

Researchregistry1025.

## Guarantor

Tetsuro Tominaga.

## Consent

Written, informed consent from the patient to publish a case report has been obtained.

## Provenance and peer review

Not commissioned, externally peer reviewed.

## Declaration of competing interest

None.
